# A randomized trial on the effects of root resorption after orthodontic treatment using pulsating force

**DOI:** 10.1186/s12903-020-01226-z

**Published:** 2020-08-27

**Authors:** Jue Wang, Ejvis Lamani, Terpsithea Christou, Peng Li, Chung How Kau

**Affiliations:** 1 Department of Orthodontics, School of Dentistry, Univeristy of Alabama at Birmingham, Room 305, School of Dentistry Building, 1919 7th Avenue South, Birmingham, AL 35294 USA; 2 School of Nursing, University of Alabama at Birmingham, Birmingham, AL USA

**Keywords:** Radiographic evaluation, Root-crown ratios, Clear aligners, Tooth movement, Cone beam computed tomography

## Abstract

**Background:**

An orthodontic device that moves teeth with pulsating force was invented and underwent a single center, controlled, clinical trial to test its safety and efficacy for treatment. The device has a custom-made thermo-plastic mouthpiece which fits over the teeth with an inflatable silicone element. A console that measures and controls the pulsating force level in real-time controls the air pressure that delivers a pulsating force. In this study, the effect of the device on root resorption during orthodontic treatment was evaluated using 3D cone beam computed tomography and compared with a control group of patients who received Invisalign treatment.

**Methods:**

Twenty-eight subjects were enrolled in the investigational arm and 15 in the control group. Subjects were followed until the average score of the mandibular and maxillary teeth achieved a Little’s Irregularity Index of 1.5 mm or less.

**Results:**

There were no adverse events reported throughout the study for either treatment arm. No clinically significant root resorption was observed for either group. The investigational device did not cause root resorption greater than the control group. Both devices produced a safety profile compared to current orthodontic techniques.

**Conclusion:**

The investigational device did not produce more root resorption than similar conventional orthodontic appliances.

**Trial registration:**

ClinicalTrials.gov, NCT03421886. Registered 12 January 2018 - Retrospectively registered.

## Background

Efforts have been continuously made in the orthodontic field to provide a more effective, esthetic, and comfortable ways of treatment for orthodontic patients. Optimum orthodontic force is critical for the effectiveness and efficiency of orthodontic treatment. However, monitoring the magnitude of the orthodontic force applied by current orthodontic appliances is difficult. Uncontrolled orthodontic force can collapse blood vessels within the periodontal ligament (PDL) leading to undesirable side effects. The cycle of PDL damage (hyalinized area formation and later undermining bone resorption) and repair is usually the main reason for prolonged orthodontic treatment and root damage.

Both in vitro and in vivo studies have shown that fully controlled pulsating orthodontic force can shorten the treatment period by potentially preventing the damage to capillary vessels within the PDL [1–4]. The effect of intermittent force versus continuous force on the amount of molar movement was examined in rats [3]. The results showed that 70% of the tooth movement was achieved within 8 h of intermitted force (33.3% relative to continuous). In addition, previous study using rats showed that an activation of force for 12 h with 12-h rest produced similar tooth movement results to those achieved when force was applied the whole day [4]. It is also indicated that there is a diurnal variation regarding the tooth’s response to orthodontic force, and that the force applied during the animal’s rest period may be more effective than that applied during its active period. Notably, when pulsating force was applied to human maxillary molars, it is found that both the rate of movement and the total movement of the treated tooth was greater than that of the controlled tooth [1].

Safety is always the first concern when introducing an innovation into the medical field. Root resorption has been known as an unavoidable side effect of orthodontic treatment and has degrees of variability. It has been shown that the resorption in the majority of teeth is less than 2.5 mm and differs in the range of 10% for different teeth [5–8]. In addition, the Malmgren index has classified severe root resorption as more than 4 mm and 1–5% of the root length [9]. The severity of root resorption during orthodontic treatment varies largely, and is closely related to multiple factors. Genetics, ethnicity, individual biological variables, and mechanical factors are common factors found relevant [10–13]. Moreover, root morphology, tooth abnormalities, trauma, and severity of malocclusion also play a part [14]. Notably, mechanical factors, such as type of appliances used, and magnitude and direction of the force applied, are associated with the severity of root resorption during orthodontic treatment [15–17]. Thus, evaluating tooth root resorption is a critical part of assessing the safety profile of an orthodontic appliance.

Due to its advantages of comfort and esthetics, clear aligners have become increasingly popular among patients seeking orthodontic treatment [18, 19]. It also presents as a safe orthodontic appliance as it is found that the prevalence and severity of external root resorption in patients with clear aligners were less than those in patients with fixed appliances [20, 21]. Thus, we designed the study to assess the external root resorption in patients treated with the innovative device which delivers pulsating orthodontic force through inflatable silicone element and compared it with that in patients treated with clear aligner. Three-dimensional cone beam computed tomography (CBCT) was used in the present study as a reliable way to measure tooth crown and root length [22, 23].

## Methods

### Study design

The study was approved by the University of Alabama at Birmingham Institutional Review Board (IRB Protocol Number F160418007). The study was designed as an open label, two-arm study with concurrent, randomized treatment and control groups. The study was conducted at a single site, the Department of Orthodontics, University of Alabama at Birmingham.

Subjects were derived from incoming patients seeking orthodontic treatment using clear aligners. When such a patient arrived, they were offered the opportunity to be enrolled in the trial. The protocol specified a sample size of 45 subjects with allocation ratio of 2:1 between the treatments (30 active and 15 control). The investigational group received the orthodontic device that delivers pulsating force (Aerodentis System, Dror Orthodesign, Jerusalem, Isreal) while the control group received clear aligners (Invisalign, Align Technology, California, USA). Subjects were followed for a total of up to 15 months or until achieving a Little’s index of < 1.5, whichever came first.Subjects will be assigned to one of the treatment arms according to randomization list, which was generated using a computerized algorithm. Randomization codes were sealed in individual envelopes per patient. The envelopes were opened only when a single randomization occurred, in order to avoid bias.

### Patient recruitment

Prior to enrolling subjects in the study, the investigator orthodontist determined through screening if the subject qualifies for the study and if the investigational device is an appropriate treatment option for the subject using the following enrollment criteria: 1) permanent dentition; 2) class l malocclusion with crowding of < 6 mm or Mild class II, class II subdivision; 3) good oral hygiene. The exclusion criteria for the study were as follows: 1) any medical or dental condition that could negatively affect study results during the expected length of the study; 2) subject is currently using any investigational drug or any other investigational device; 3) subject plans to relocate or move during the treatment period; 4) allergic to acetaminophen (use of aspirin or non-steroidal anti-inflammatory drugs is excluded for subjects while on the study); 5) use of bisphosphonates (osteoporosis drugs) during the study; 6) pregnant females; 7) subjects that are likely unwilling to be compliant with device use, as determined by the questionnaire for compliance. All subjects enrolled in the study met all the enrollment criteria.

### Investigational device

**Fig. 1:**
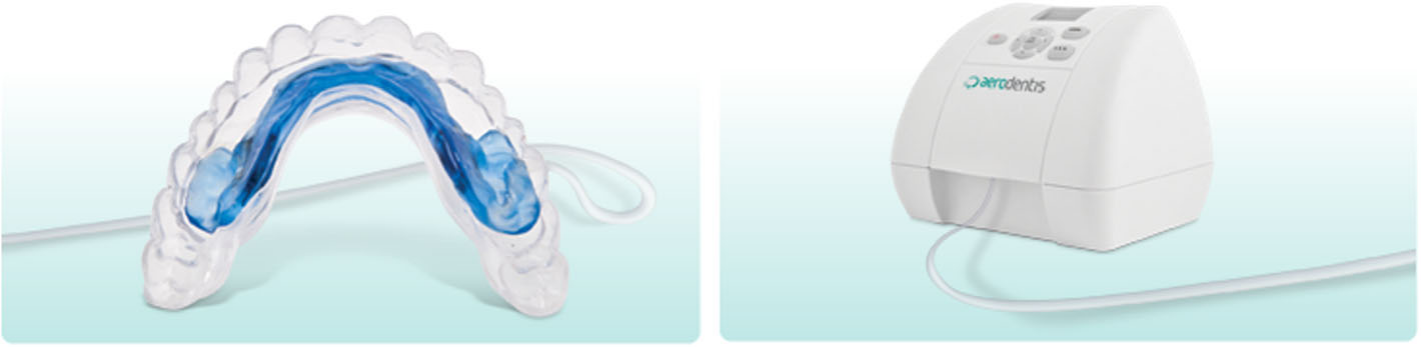
A thermo-plastic mouthpiece with an integrated inflatable silicone element and the console

The investigational device [24] was recently approved by Food and Drug Administration of United States (FDA #: K192069). It consists of two main components (Fig. 1):

1. A thermo-plastic mouthpiece with an integrated inflatable silicone element. The mouthpiece is specifically designed for the subject and produced from orthodontic sheets by vacuum forming technology using CAD/CAM (computer-assisted design/computer assisted manufacturing) and 3D imaging.
2. A small easy-to-operate console with a user-friendly interface. It houses the electronics, air pump system and pressure sensor that measures and controls the electronic pulsating force level in real-time. An easy to use smart card monitors subject’s compliance in treatment.

The inflatable element replaces the wires and brackets of traditional metal braces. Movement is achieved by applying pneumatic force on the buccal or and lingual surfaces of the teeth being treated.

### Measuring root resorption by CBCT

Previous studies have shown that the CBCTs are good sources to measure root resorption [25, 26]. All CBCT images were taken using the Kodak 9300 CBCT (Carestream Dental LLC, USA) machine. The CBCT scan of a patient was taken with a voxel resolution of 0.3 mm. Each scan was saved in a Dicom file format. Two CBCT scans were taken (one at each time point) before orthodontic treatment (T1) and at the completion of tooth alignment (T2). As the pulsating force of the investigational device only applies to the maxillary and mandibular anterior teeth, measurements were performed on 12 teeth (maxillary and mandibular canines and incisors). All CBCT scans were approved by IRB and consented by the participants.

**Fig. 2:**
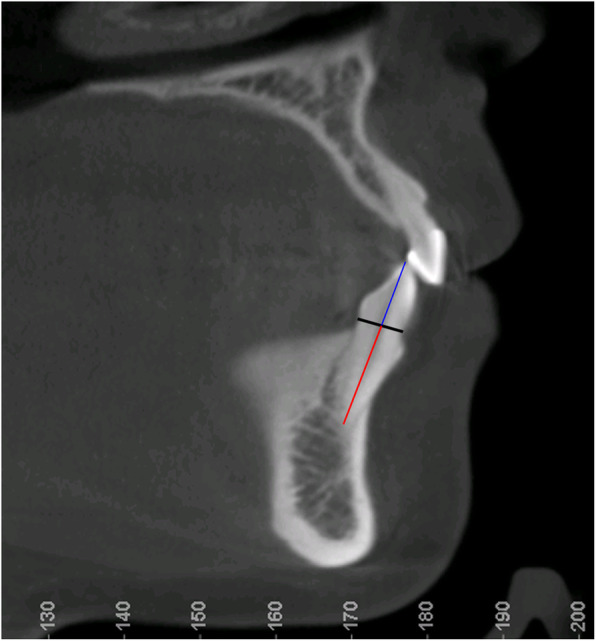
Measurement of tooth crown length (blue line) and root length (red line) on CBCT

Crown and root lengths were measured and analyzed by using a method developed by Lind [27] and modified by Holtta et al. [28]. Midpoint was visually determined along a line bisecting the buccal and lingual cemento-enamel junctions (CEJ) (Fig. 2). All the teeth measured were single-rooted teeth as only anterior teeth were included. Thus, each root was measured from the apex to the corresponding midpoint. All crown heights were measured from the CEJ midpoints perpendicular to the incisal/occlusal reference line (formed tangent to incisal edge or buccal cusps). Root resorption for an individual tooth was assessed using the following formula:

$$ {\displaystyle \begin{array}{l} EARR={Root}_0\frac{Crown_f}{Crown_0}-{Root}_f\\ {} EARR\ Ratio=\frac{EARR}{Root_0}\end{array}} $$

Where EARR is external apical root resorption, f is the final measurement, and 0 is the baseline measurement.

### Teeth movement assessment (LII)

Teeth movement was assessed by measuring Little’s Index at 7 weeks and every 2 months thereafter. Tooth misalignment was measured in millimeters by means of a caliper and Little’s Index was calculated.

### Statistics

The sample size was calculated based on previous root resorption studies with similar study designs [20, 29, 30]. When the sample sizes in the groups are 15 and 30, a two-group large-sample normal approximation test of proportions with a one-sided 0.05 significance level will have 80% power to reject the null hypothesis that the test and the standard are not equivalent.

The patients’ demographic and clinical characteristics were summarized as mean and standard deviation (SD) for continuous variables or frequency and proportion for categorical variables. EARR and EARR ratio were summarized at both tooth and patient level. The group comparison of EARR or EARR ratio at tooth level was conducted using Wilcoxon test while patient level comparison was conducted with a random effect model to take the clustering effect (multiple teeth within one patient) into account. The false discovery rate (FDR) was used for the multiple comparison correction. The correlation between LII and patient level EARR or EARR ratio was evaluated using Spearman correlation coefficient. *P* < 0.05 or FDR < 0.05 given multiple comparison was considered as statistically significant. All the analysis was conducted using SAS 9.4 (Cary, NC).

## Results

### Subjects

A total of 36 investigational and 15 control subjects were enrolled into the trial from August 2016 through June 2018. However, 8 of the investigational subjects were excluded after consent as they chose not to participate further or did not conform to the compliance regime. Of the remaining 28 investigational and 15 control subjects, 5 investigational subjects and 2 control subjects did not complete the trial. For the investigational subjects, failure to complete the treatment were due to balloon leakage issues, technical issues with the console, and mouthpiece retention issue. The subjects withdrew from the control group stated that they preferred fixed appliances. All subjects that remained in the trial successfully achieved the primary endpoint. No subjects withdrew as a result of adverse events.

Demographics of the participants were shown in Table 1. Subject age ranged from 12 to 65, with the average age being 31. The majority of subjects were female (86%), consistent with most aesthetic devices.

**Table 1:** Baseline characteristics of participants in the two groups

	Aerodentis (*N* = 28)	Control (*N* = 15)
Age (Years) (Mean ± SD)	30.6 ± 12.0	32.3 ± 16.4
Sex (N, %)		
Male	4 (14.3%)	5 (33.3%)
Female	24 (85.7%)	10 (66.7%)

### Root morphology measurements at T2 compared to T1

The safety endpoint was the incidence of adverse events, whether or not treatment related, and the amount of root resorption, as measured by comparing x-rays before and at the end of tooth alignment. There were no adverse events reported for either the investigational or control devices.

The EARR for the investigation subjects (averaged across all teeth) was 0.16 ± 0.172 mm and 0.071 ± 0.045 mm for control subjects with resorption ranging from 0 to 0.84 mm for the investigational group and 0.0–0.2 mm for the control group (Table 2). After multiple testing correction, no significant difference was observed for all teeth. The analysis for EARR Ratio yielded similar results, with the root resorption ratio (averaged across all teeth) 1.238% (± 1.326%) in the investigational group and 0.511% (± 0.359%) in the control group (Table 3). In any event, the extent of root resorption is minor and not expected to be clinically relevant as it is in the range of normal root resorption [26, 31] and less than 1 mm, which is the generally accepted limit.

**Table 2:** Root resorption measurement: EARR change from baseline in the two groups

EARR (mean ± SD, mm)	*Aerodentis*	*Control*	*P-value*	*FDR*
Maxillary right canine	0.21 ± 0.22	0.06 ± 0.04	0.0177	0.1011
Maxillary right lateral incisor	0.12 ± 0.11	0.08 ± 0.03	0.203	0.2436
Maxillary right central incisor	0.12 ± 0.10	0.09 ± 0.04	0.3094	0.3094
Maxillary left central incisor	0.11 ± 0.16	0.04 ± 0.02	0.1129	0.1694
Maxillary left lateral incisor	0.20 ± 0.17	0.09 ± 0.05	0.0292	0.1011
Maxillary left canine	0.13 ± 0.13	0.09 ± 0.06	0.2614	0.2852
Mandibular right canine	0.21 ± 0.22	0.09 ± 0.06	0.0513	0.1025
Mandibular right lateral incisor	0.14 ± 0.16	0.07 ± 0.04	0.1303	0.1737
Mandibular right central incisor	0.17 ± 0.19	0.06 ± 0.05	0.0561	0.1025
Mandibular left central incisor	0.13 ± 0.09	0.07 + 0.04	0.0317	0.1011
Mandibular left lateral incisor	0.15 ± 0.16	0.06 ± 0.03	0.0598	0.1025
Mandibular left canine	0.23 ± 0.26	0.07 ± 0.06	0.0337	0.1011

*SD* Standard deviations, *FDR* False discovery rate

**Table 3:** Root resorption measurement: EARR ratio change from baseline in the two groups

EARR Ratio (mean ± SD, %)	*Aerodentis*	*Control*	*P-value*	*FDR*
Maxillary right canine	1.40 ± 1.46	0.29 ± 0.20	0.0113	0.0714
Maxillary right lateral incisor	0.90 ± 0.82	0.55 ± 0.19	0.1258	0.1510
Maxillary right central incisor	0.92 ± 0.83	0.61 ± 0.28	0.194	0.1940
Maxillary left central incisor	0.80 ± 1.20	0.26 ± 0.16	0.1074	0.1432
Maxillary left lateral incisor	1.56 ± 1.33	0.62 ± 0.37	0.0192	0.0714
Maxillary left canine	0.97 ± 0.99	0.53 ± 0.38	0.141	0.1538
Mandibular right canine	1.57 ± 1.67	0.68 ± 0.76	0.0866	0.1432
Mandibular right lateral incisor	1.25 ± 1.55	0.51 ± 0.31	0.0975	0.1432
Mandibular right central incisor	1.61 ± 1.91	0.54 ± 0.37	0.06	0.1276
Mandibular left central incisor	1.20 ± 0.94	0.56 + 0.24	0.0221	0.0714
Mandibular left lateral incisor	1.15 ± 1.18	0.53 ± 0.26	0.0638	0.1276
Mandibular left canine	1.56 ± 1.67	0.45 ± 0.34	0.0238	0.0714

*SD* Standard deviations, *FDR* False discovery rate

### Correlation between LII and root resorption

LII was calculated for both maxilla and mandible for all the patients; for the experiment group, this ranged from 0.5–14.45 mm and, for the control group, 0.7–13.5 mm, which illustrates the degree of crowding. Positive correlation was found between LII and the severity of root resorption but not statistically significant, possibly due to small sample size (Table 4).

**Table 4:** Correlation between Little’s Irregularity Index (LII) and root resorption

	Mean EARR	Mean Ratio	LII
For all patients and mandibular site			
Mean EARR	1	0.95692	0.30198
		<.0001	0.1423
Mean Ratio	0.95692	1	0.36045
	<.0001		0.0767
LII	0.30198	0.36045	1
	0.1423	0.0767	
For all patients and maxillary site			
Mean EARR	1	0.95308	0.2836
		<.0001	0.1897
Mean Ratio	0.95308	1	0.33696
	<.0001		0.1159
LII	0.2836	0.33696	1
	0.1897	0.1159	

## Discussion

No adverse events were reported throughout the study for either study arm, suggesting that the device was safe for clinical purpose. Although the mean EARR for the investigation subjects was larger than that for the control group, the difference was not statistically significant. As a result, the investigational device has a comparable safety profile to the predicate from the perspective of root resorption.

Both panoramic radiographs and CBCT were used in the earlier studies to assess tooth root length [20, 21, 26, 30]. Currently, CBCT has been widely used in orthodontic treatment as an important tool for determining impacted tooth location [32], facial asymmetry [33], supernumerary teeth, temporomandibular joint (TMJ) pathology [34], airway construction, as well as for surgical planning [35, 36]. Previous study has shown that the tooth length and root length measured by CBCT has no significant difference from the actual tooth length, indicating that CBCT scan provides enough accuracy for tooth crown and root length measurement [22].

Tooth root resorption related to orthodontic treatment mainly occurs in the anterior teeth [37, 38]. Since the inflatable silicone element in the mouthpiece of the investigational device extends only in the anterior region, root measurements were made to the area of anatomy. Previous studies on tooth root resorption also focused on the anterior teeth for practical purpose, as well as for better accuracy of measurement [37, 39].

In the present study, both tooth root length and crown height were measured and the formula for EARR and EARR ratio were used. This method of measurement was used according to previous studies [39, 40] and chosen specifically to avoid any magnification error when analyzing CBCT scans. The formula was used on the basis that tooth crown height remains the same before and after the treatment. As a result, the difference of root length between the two time points represented the true root resorption amount.

From previous studies, it is generally considered that EARR of 1 mm or more during any 12-month period of active treatment is clinically significant [41, 42]. In the present study, the mean EARR for the investigation subjects was 0.16 ± 0.17 mm and was 0.07 ± 0.05 mm for control subjects. Thus, the extent of root resorption for both groups were not clinically significant. Previous study has reported that the degree of EARR for fixed appliances is about 1.67 ± 0.64 mm for maxillary central incisors [40]. Another recent study has shown an average of 1.12 ± 1.34 mm root resorption for fixed appliance [20]. Thus, our data is in accordance with the previous study that the severity of EARR in patients using clear aligners was less than those using the fixed appliance. In addition, the severity of EARR in the investigational group was shown to be less than what has been reported with the fixed appliance, indicating that the fully controlled pulsating force may be more optimal than the force delivered by the fixed appliance.

Little’s Index is the summation of the distances of the tooth contact points along the occlusal axis. It is used to assess anterior crowding and reflects rotation irregularities and displacement. In the present study, positive correlation was found between LII and the severity of root resorption. This is consistent with the previous study that duration of treatment and the horizontal displacement of the incisor apices were significantly associated with root resorption [43]. It is generally agreed that the larger the Little’s index value, more anterior crowding is present. And it also takes a longer treatment time to move teeth. However, the positive correlation seen in this study was not statistically significant, possibly due to small sample size in the current study.

### Limitations

Due to the obvious differences between the investigational and control devices, the users could not be blinded to their treatment arm. However, the investigators were blinded when doing the measurement for root resorption and the Little’s Index.

The planned sample size (30 in the investigational group and 15 in the control group) was decided according to the power analysis, with α = 0.05 and 80% power. However, there were 5 investigational subjects and 2 control subjects who did not complete the trial. Although most participants (80%) in the investigational group stated that they were very satisfied or satisfied with the device (data not shown), occasional technical issues, which were to be expected for a new technology, arose and led to the failure of some participants to finish the trial. We expect that new improvement be made to the device based on the data collected from this study.

## Conclusion

This results from this study suggest that the investigational device delivering pulsating force is safe and can be used clinically. No clinically significant root resorption was observed for either the investigational or the control group, and investigational device did not cause root resorption greater than the control group.

## Data Availability

The data sets used and analyzed during the current study are available from the corresponding author upon reasonable request.

## Abbreviations

PDL: Periodontal ligament
CBCT: Cone beam computed tomography
CAD/CAM: Computer-assisted design/computer assisted manufacturing
EARR: External apical root resorption
CEJ: Cemento-enamel junctions
LII: Little’s index
SD: Standard deviation
FDR: False discovery rate
TMJ: Temporomandibular joint
